# Science Is About Thinking: How Can We Protect Thinking Time in a Distracted Digital World?

**DOI:** 10.3390/brainsci16070677

**Published:** 2026-06-27

**Authors:** Wissem Dhahbi, David B. Pyne, Ismail Dergaa, Daniel Zeitouny, Patrick Müller, Abdelfatteh El Omri, Karim Chamari, Helmi Chaabene

**Affiliations:** 1 Research Unit “Sport Sciences, Health and Movement”, Institut Supérieur de Sport et de l’Éducation Physique du Kef, Université de Jendouba, Le Kef 7100, Tunisia; wissem.dhahbi@gmail.com; 2 Training Department, Police College, Qatar Police Academy, Doha 7157, Qatar; 3 Research Institute for Sport and Exercise, University of Canberra, Canberra, ACT 2617, Australia; david.pyne@canberra.edu.au; 4 Tunisian Research Laboratory “Sports Performance Optimization”, National Center of Medicine and Science in Sports (CNMSS) LR09SEP01, Tunis 1004, Tunisia; ismael.dergaa@hotmail.fr; 5 Surgical Research Section, Department of Surgery, Hamad Medical Corporation, Doha 3050, Qatar; dzeitouny@hamad.qa (D.Z.); aelomri@hamad.qa (A.E.O.); 6 Department of Cardiology and Angiology, University Hospital Magdeburg, Otto-von-Guericke University Magdeburg, 39120 Magdeburg, Germany; patrick.mueller@med.ovgu.de; 7 Clinical Advancement Department, Hamad Medical Corporation, Doha 3050, Qatar; 8 Research Department, Naufar Center, Doha 3263, Qatar; karim.chamari@naufar.qa; 9 Higher Institute of Sport and Physical Education, ISSEP Ksar Saïd, Manouba University, Tunis 2010, Tunisia

**Keywords:** attention management, cognitive neuroscience, default mode network, mindfulness, neuroplasticity, professional burnout, sleep hygiene, work–life balance

## Abstract

**Highlights:**

**What are the main findings?**

- Digital distractions and media multitasking are associated with the disruption of the coordinated dynamics between the executive control network and the default mode network, and with neuroplastic changes that may impair sustained attention.
- The constant fragmentation of attention in modern research environments degrades the specific cognitive operations required for complex problem-solving, deep analysis, and creative scientific insight.

**What are the implications of the main findings?**

- Protected thinking time must be recognized not merely as a workplace preference, but as a critical neurobiological precondition necessary to preserve cognitive function and advance high-quality scientific discovery.
- Research institutions should implement evidence-based, multi-level strategies, spanning individual practices, organizational policies, and technological boundaries, to safeguard uninterrupted cognitive intervals and move beyond purely quantitative productivity metrics.

**Abstract:**

Background and Aims: Rapid digital transformation has generated pervasive attentional disruption in research and professional settings, raising the question of how the temporal conditions that support deep scientific thinking can be preserved. Our narrative review aimed to (i) synthesize neurobiological evidence on the mechanisms through which task-irrelevant digital interruption impairs deep thinking; (ii) discuss the conditions required for deep thinking and the potential threats posed by contemporary developments, including generative artificial intelligence-related cognitive offloading; and (iii) elaborate evidence-based, multi-level recommendations for research institutions. Methods: Targeted searches of PubMed, Google Scholar, and Web of Science (January 2010–September 2025) were conducted using terms spanning attentional neuroscience, digital distraction, neuroplasticity, and cognitive performance, supplemented by forward and backward citation tracking. Peer-reviewed empirical studies, meta-analyses, and theoretical frameworks addressing neurobiological mechanisms of sustained attention and the cognitive effects of digital interruption in professional and/or research settings were included. Results and Interpretation: Deep thinking and protected thinking time are treated as distinct constructs: the former as a sustained, integrative cognitive process supported by coordinated executive control and default mode network activity, the latter as uninterrupted temporal intervals within which that process can occur. Repeated engagement with task-irrelevant digital stimuli is associated with cortico-striatal strengthening and prefrontal-parietal under-consolidation, producing a plasticity paradox in which attentional fragmentation becomes self-reinforcing. The emergence of generative artificial intelligence introduces a qualitatively distinct threat through voluntary cognitive offloading, which reduces deep engagement independently of attentional distraction. Conclusions: Evidence-based strategies spanning individual, team, organizational, technological, and assessment levels are available to preserve protected thinking time. Direct evidence linking these intervals to specific research-impact outcomes remains limited, and institutional interventions should be prospectively evaluated.

## 1. Introduction

Contemporary digital environments pose cognitive challenges for knowledge workers, here defined as professionals whose primary output is the production, analysis, or transformation of information rather than physical goods [1]. Researchers represent a particularly vulnerable subgroup due to the intensive cognitive demands of scientific inquiry, including processing complex data, integrating findings across disciplines, and translating research into actionable recommendations [2]. The unwavering pursuit of efficiency and the widespread adoption of digital tools have resulted in a significant decline in a decisive aspect of scientific and medical investigations: the time dedicated to deep thinking [2]. Digital communication technologies have evolved from the internet and email to multi-media messaging apps (e.g., WhatsApp) and now include artificial intelligence (AI) chatbots and new tools for literature searches [3,4]. While these innovations have revolutionized scientific collaboration and information sharing, they have created a significant contradiction. While these technologies enable quicker research processes, they concurrently reduce the time available for deep concentration and reflection [2]. Recent empirical studies have documented how media multitasking affects cognitive processing, with research showing consistent links between heavy media multitasking and decreased cognitive performance across various domains [5]. Digital distraction encompasses constant interruptions from various technological sources, including smartphones, social media, email, and instant messaging platforms. A conceptual distinction is necessary at the outset. Digital tools are not uniformly disruptive. Task-aligned technologies, including literature databases, reference managers, statistical environments, and domain-specific AI assistants invoked in service of the current cognitive task, can extend working memory and accelerate verification, supporting rather than displacing deep thinking. This distinction has a neurocognitive basis: mechanical and digital technologies recruit dissociable neural pathways, with digital (opaque) tools engaging circuits that differ from those underlying transparent tool use and deliberate reasoning [6,7]. An integrated account of technological cognition specifies that certain cognitive operations, including goal maintenance and integrative inference, are particularly vulnerable to displacement by digital task-irrelevant streams [8], and that the opacity characteristic of digital tools compounds this vulnerability by reducing the metacognitive signals that normally regulate cognitive engagement [9]. Task-irrelevant digital streams, including push notifications, instant messaging, social media, and entertainment platforms, fragment attention through repeated involuntary orienting and do not contribute to the immediate cognitive goal. The remainder of this review addresses the latter category, while recognising that the boundary is determined by use context rather than by the technology itself. Specifically, the constant connectivity and multitasking facilitated by digital technologies have been shown to alter brain plasticity and impair cognitive functions such as attention, potentially affecting performance across various life domains [10].

The influence of this proliferation of digital communication tools on the quality of scientific output is very worrying. Although scientific output has increased quantitatively, recent analyses of publications and patents suggest that their propensity to shift fields in new directions has declined [11]. This decline reflects complex interactions between technological disruption, economic pressures on academic institutions, policy-driven productivity metrics, and changing reward structures that prioritize publication quantity over translational impact [12]. This is reflective of a diminished influence of science on human’s daily life, a concerning trend that warrants significant attention [13]. Newport’s “Slow Productivity” framework challenges the prevailing belief that productivity must continuously increase, proposing instead a deliberate strategy emphasizing qualitative depth over quantitative output—a perspective particularly relevant within the hyperproductive academic publishing environment [14]. This viewpoint is especially important in today’s research landscape, when there is a strong emphasis on the quantity of publications [15]. Researchers are expected to fulfil critical tasks, including analysing complex mechanisms, integrating multidisciplinary perspectives, and translating findings into practical recommendations or interventions. These tasks require remarkable cognitive demands that cannot be met without substantial protected thinking time. A critical examination of research practices suggests that adequate time allocation for analysis and interpretation may enhance the quality of findings, although specific evidence for a direct correlation between analysis duration and research impact remains limited [16].

“Protected thinking time” encompasses uninterrupted periods essential for complex cognitive tasks. This time is required for activities such as reflecting on existing phenomena, designing new experiments and work practices, processing data, examining literature, and/or writing scientific articles [17]. Nevertheless, this essential feature is frequently overlooked and hardly quantified in employment practices [18]. The absence of recognition and protection of thinking time within institutional and organisational frameworks is a notable obstacle for researchers and practitioners seeking to develop and uphold the standard of their work. Research on information processing demonstrates that the brain requires uninterrupted time to engage the default mode network (DMN), which is essential for creative connections and scientific breakthroughs [19,20]. This narrative review addresses one central question: how can the temporal conditions that allow deep thinking be protected in a digitally saturated research environment? We define deep thinking as the sustained, integrative cognitive process supported by the coordinated activity of executive control and default mode networks. We define protected thinking time as the bounded interval, free from external interruption, within which deep thinking can occur. The two are related but not equivalent; deep thinking is the process, protected thinking time is the precondition.

Digital distraction is not merely an individual behavioural problem but a systemic one: it is simultaneously produced by technological design, organisational reward structures, and institutional productivity norms that collectively erode the conditions for sustained cognitive work [11,12]. Understanding this multi-level aetiology is necessary to develop interventions that are architecturally coherent rather than individually palliative. Against this background, our narrative review addresses three questions: (i) What are the known neurobiological mechanisms by which task-irrelevant digital interruption impairs deep thinking? (ii) What neurobiological and temporal conditions are required for deep thinking to occur, and what contemporary developments, including generative AI cognitive offloading, threaten those conditions?? (iii) What evidence-based strategies can research institutions implement across individual, team, organisational, technological, and assessment levels to preserve protected thinking time, and what are the current evidentiary limits of such recommendations? These questions are returned to in the Conclusion.

### Review Methodology

This narrative review aimed to: (i) synthesise neurobiological evidence on the mechanisms through which task-irrelevant digital interruption impairs deep thinking; (ii) characterise the individual, organisational, and technological conditions that constitute protected thinking time; and (iii) elaborate evidence-based, multi-level recommendations for preserving uninterrupted cognitive intervals in research settings. Literature synthesis covered digital distraction, cognitive neuroscience, and protected thinking time in research environments. Literature identification employed targeted searches of PubMed, Google Scholar, and Web of Science databases (January 2010–September 2025), supplemented by forward and backward citation tracking of seminal studies. Search terms combined cognitive domains (attention, neuroplasticity, default mode network, executive function, memory consolidation), digital environment descriptors (multitasking, digital distraction, technostress, attention fragmentation, generative artificial intelligence, cognitive offloading, technological cognition), and outcome variables (productivity, creativity, research quality, burnout). Inclusion criteria comprised peer-reviewed empirical studies, systematic reviews, meta-analyses, and theoretical frameworks published in English that addressed neurobiological mechanisms of sustained attention, the cognitive effects of digital interruption, or evidence-based interventions for protecting uninterrupted cognitive work. Studies were excluded if they addressed distraction exclusively in clinical or paediatric populations without relevance to the research or professional knowledge-worker context. Selection prioritized peer-reviewed empirical studies, meta-analyses, and theoretical frameworks addressing neurobiological mechanisms underlying sustained attention and the impact of digital interruptions on cognitive performance. Inclusion prioritized recent English-language publications while also integrating foundational neuroscience works that defined the underlying mechanisms. WD and HC conducted the literature search and synthesis; all authors contributed to conceptual framing and evidence evaluation. Given the narrative scope and word constraints of this narrative review format, we employed a selective rather than exhaustive approach, focusing on high-impact studies that illuminate neurobiological mechanisms and evidence-based interventions. This selective approach, aligned with narrative review methodology [21,22], prioritises high-impact mechanistic and interventional evidence over exhaustive retrieval, and differs from a systematic review in that it does not employ formal screening protocols or eligibility-based exclusion at the article level.

## 2. The Neurobiological Basis of Scientific Thought

Neuroimaging research reveals that digital distraction affects cognitive mechanisms underlying sustained attention, with implications extending from scientific thinking to educational performance and workplace productivity across diverse populations [10]. The human brain facilitates both conscious deliberation and subconscious processing, both vital components of scientific inquiry and creative problem-solving. Deliberate attention is crucial for problem-solving and ideation, as it requires deliberate thought [19]. Engagement of the DMN during periods of unconstrained rest supports memory consolidation, off-task information processing, and the formation of remote associations that underlie creative insight [20,23]. These neurobiological processes operate consistently across life domains, suggesting that protecting cognitive function benefits not only scientific work but also educational achievement, professional performance, and general well-being [24].

Functional magnetic resonance imaging and electroencephalography studies demonstrate that the dynamic interaction between focused thinking (characterized by executive network activation) and mental relaxation (characterized by DMN engagement) is crucial for creating new concepts and association of ideas that underpin advances in science, with measurable increases in alpha wave coherence during creative insight moments [25,26]. Neural networks require continuous periods free of distractions to effectively process information, establish connections, and produce novel ideas [25]. This neural process is vital across scientific disciplines, where progress requires integrating multiple complex systems with behavioral factors and performance metrics across diverse populations [27]. These considerable cognitive demands cannot be adequately met through fragmented thinking. Beaty et al. [28] reported that high-creativity individuals show enhanced functional connectivity between executive control and DMN during creative tasks. Generating new ideas, analysing information, and combining knowledge from various disciplines require long periods of focused mental effort [14]. The achievement of deep thinking is difficult during disjointed time periods, emphasising the importance of developing settings that cultivate both conscious and implicit (unconscious) thoughts [17,29]. Presumably, these settings would span professional practice, academic environments, and the general community.

## 3. Neuroplasticity as the Central Mechanism Linking Digital Distraction to Impaired Deep Thinking

Neuroplasticity, the experience-dependent reorganisation of synaptic structure and function, provides the principal mechanistic link between repeated digital interruption and degraded deep thinking. Cross-sectional evidence indicates that habitual engagement with short, reward-contingent stimuli is associated with cortico-striatal strengthening tuned to rapid stimulus switching, and with reduced consolidation of long-range prefrontal-parietal networks supporting integrative cognition [10,30]. This asymmetry produces what may be termed a plasticity paradox: the same adaptive property that allows expert cognition to be acquired also entrenches the cognitive profile elicited by the dominant daily stimulus environment. Cross-sectional neuroimaging studies in heavy media multitaskers have reported associations between multitasking frequency and reduced grey-matter density in the anterior cingulate cortex, a region central to conflict monitoring and attentional control [10]; yet, the directionality of this association remains contested [10]. Functionally, heavy multitaskers display weaker top-down attentional filtering and increased susceptibility to irrelevant stimuli [5]. These adaptations matter for scientific work because the cognitive operations that distinguish high-impact research, hypothesis generation, integration across studies, and the recognition of weak signals in complex data, depend precisely on the networks most vulnerable to this displacement. From the perspective of technological cognition, digital opacity, the property whereby digital tools obscure the computational operations they perform from the user, may amplify these effects by suppressing the metacognitive monitoring that normally enables attentional self-regulation [9]. Protected thinking time may accordingly be understood not merely as a productivity preference but as a candidate neurobiological precondition: the temporal substrate within which the prefrontal-parietal networks underlying deep thinking could be re-engaged and, over time, consolidated.

## 4. Challenges to Effective Cognitive Processing in Research

### 4.1. The Detrimental Effects of Time Constraints and Multitasking

Beyond the immediate performance cost, repeated interruption produces what has been described as attention residue, whereby thoughts about a prior task persist and degrade performance on the current one [14]. Resumption lags after digital interruption have been documented in workplace studies [31], and laboratory work links heavier media multitasking to attention lapsing and subsequent memory failure [32]. Cross-sectional and observational studies have reported structural and functional associations between sustained digital multitasking and prefrontal alterations [10], though causal directionality has not been established yet.

The continuous need to stick to deadlines and generate research at an elevated rate could harm the standard and thoroughness of scientific research and professional practice. When researchers and practitioners constantly handle numerous tasks while working within strict time limits, their ability to do serious, focused thinking is impaired. Converging evidence from experimental and narrative-review work indicates that multitasking impairs cognitive performance, with documented decrements in accuracy and increases in completion time across a range of laboratory paradigms [5,33]. In research settings, the multitasking burden is intensified by multiple responsibilities. Researchers must frequently shift between various duties, data collection and analysis, and communication with multidisciplinary team members [34].

In addition, chronic stress, which is frequently linked to an overwhelming workload and time constraints, may adversely affect mental capacity and innovative thinking [35]. Studies indicate that stress can result in a decline in attention span, a reduction in problem-solving skills, and an increase in susceptibility to cognitive biases [36,37]. While we acknowledge that multitasking and time limitations are inevitable, individuals can mitigate their impact by recognizing their negative consequences and creating, at least for selected periods during the day/week, a safer protected environment that fosters creative thinking [19].

### 4.2. The Impact of Digital Distraction

Knowledge workers navigate complex communication ecosystems encompassing academic, institutional, and external networks. This creates a particularly fragmented communication landscape that exceeds the already substantial digital burden faced by researchers in laboratory-only disciplines. While designed to enhance connectivity and productivity, these tools often serve as sources of continuous distraction. This constant stream of digital stimuli can fragment attention, impeding an individual’s ability to engage in sustained, deep cognitive work essential for scientific breakthroughs [38]. The effects of digital distraction are widespread, impacting occupational safety and health in the workplace [39] and influencing cognitive development in children [40]. Research by Stothart et al. [41] demonstrated that smartphone notifications disrupt attention and cognitive performance during demanding tasks, with cumulative effects on cognitive capacity throughout the workday. Digital information flow fragments attention while reinforcing immediate reward-seeking patterns through multiple psychological mechanisms including novelty bias, intermittent reinforcement, and social validation, potentially compromising sustained cognitive work [42]. Earlier studies [10,30] have documented specific implications of digital distraction on both, physical and mental health. Specifically, physical effects include digital eye strain, poor posture, and musculoskeletal disorders [43], while mental health impacts encompass increased anxiety, decreased attention span, and impaired emotional regulation [44]. The compulsion to respond immediately to every incoming digital communication, regardless of its urgency, mirrors an irrational obligation to process all incoming correspondence the moment it arrives [45]. This pattern, now well-documented in professional knowledge-worker populations, directly reduces the cumulative duration available for uninterrupted cognitive work.

**Figure 1:**
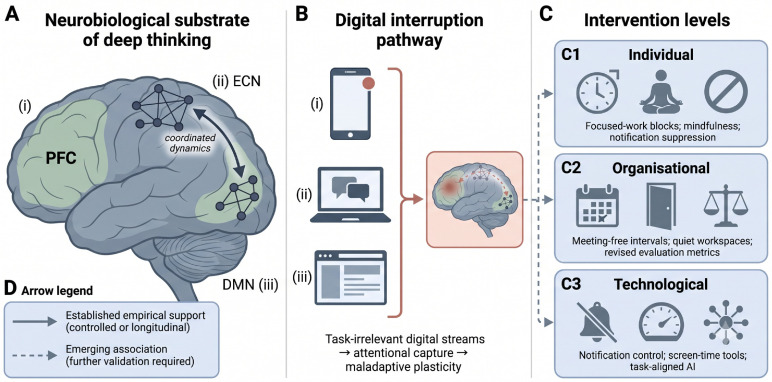
Mechanistic framework linking digital interruption to impaired deep thinking and corresponding intervention levels. (**Panel** (**A**)): neurobiological substrate of deep thinking, comprising coordinated activity of the executive control network and the default mode network (DMN), supported by prefrontal cortex (PFC) integration. (**Panel** (**B**)): digital-interruption pathway, whereby task-irrelevant notifications and media multitasking degrade top-down attentional filtering and, through repeated exposure, drive maladaptive neuroplastic remodelling. (**Panel** (**C**)): intervention levels addressing the pathway, organised as individual, organisational, and technological strategies. (**Panel** (**D**)): arrow legend, where solid arrows denote relationships supported by controlled experimental or longitudinal evidence and dashed arrows denote emerging associations requiring further empirical validation. Abbreviations: prefrontal cortex (PFC); executive control network (ECN); default mode network (DMN); artificial intelligence (AI).

Recent studies [46,47] have highlighted the detrimental effects of constant digital interruptions on cognitive performance. Moreover, the phenomenon of “digital multitasking”—attempting to engage with multiple digital streams simultaneously—has been shown to reduce the quality of work output and increase stress levels among knowledge workers [5]. The concept of “technostress”, defined as the stress experienced by individuals due to their use of information and communication technologies, has gained prominence in the recent literature [48]. A systematic review by Rademaker et al. [49] reported that technostress is associated with reduced job satisfaction, decreased productivity, and increased burnout among professionals, including academic researchers (Figure 1). A qualitatively distinct challenge has emerged with the proliferation of generative AI (GenAI) tools. Unlike push notifications or social media, which disrupt attention through involuntary orienting, GenAI platforms invite the voluntary offloading of substantive cognitive operations, including hypothesis generation, literature synthesis, and argumentation, to automated systems [6,7]. This cognitive offloading differs mechanistically from distraction: it does not fragment attention but may progressively reduce the depth of engagement that produces expertise and insight [8]. The effective use of LLMs in research, therefore, requires deliberate collaborative critical practices, in which researchers maintain authorial and analytical agency, interrogate AI-generated outputs against primary sources, and reserve generative cognitive work for unassisted protected thinking intervals.

## 5. The Benefits of Protecting Thinking Time

Devoting specific time for thinking can yield substantial advantages for researchers. Researchers can utilize undisturbed reflection to explore novel ideas, identify potential gaps in study rationales, methodology, analysis and/or data interpretation, and cultivate unique insights and applications for their work [50]. Research has demonstrated that allocating dedicated time for thinking can improve creativity, problem-solving skills, and the overall quality of scientific research [51]. Moreover, consistent intervals of introspection can assist researchers in preserving a feeling of objectivity and preventing excessive fixation on immediate objectives. Researchers can enhance their decision-making and contribute to the long-term progress of their profession by carefully reflecting on the wider ramifications of their studies and activities. The specific case of serendipity might be misleading to some, but even if described as “unplanned fortunate discovery”, serendipity ideas come from thoughtful process, even if this might be unconscious [52].

## 6. The Role of Sleep and Cognitive States

Sleep is mechanistically central to the cognitive operations underlying deep thinking, and its function here is not interchangeable with general sleep hygiene. Three pathways are directly relevant. First, slow-wave sleep supports systems-level memory consolidation through hippocampal–neocortical dialogue, stabilising new representations and enabling their integration with prior knowledge [53]. Second, REM sleep facilitates the recombination of remote associative material, a substrate of creative insight [54]. Third, sleep restriction produces disproportionate impairment of prefrontal cortex function, with measurable decrements in attentional control, working memory, and cognitive flexibility [55], the same operations on which deep thinking depends. Glymphatic clearance of metabolic by-products during sleep provides a fourth, longer-horizon mechanism with implications for cognitive resilience [56]. Within the present framework, sleep therefore acts on the quality of deep thinking rather than on the quantity of protected thinking time, and chronic sleep curtailment in research environments should be treated as a mechanistic threat to scientific output rather than as a lifestyle issue [57,58].

The bidirectional relationship between cognitive function and sleep quality creates both risk and opportunity for scientists, digital distraction disrupts sleep architecture through increased arousal and blue light exposure, while protected thinking time practices may enhance sleep quality through reduced cognitive and emotional activation [58]. Aside from sleep, engaging in mindfulness meditation and creative imagery can also improve cognition [56].

## 7. Discussion

### 7.1. Strategies for Implementing Protected Thinking Time and Preserving Cognitive Sanctuary

We propose evidence-based strategies targeting documented cognitive disruption mechanisms while supporting neural processes underlying scientific thinking. A list of potential strategies for promoting critical thinking [59] is displayed in Table 1. Strategies operate at five complementary levels. Individual practices address attention regulation and cognitive scheduling. Team-based practices address the social-coordination layer through which interruption is generated and propagated, including asynchronous communication norms and shared deep-work intervals. Organisational practices address structural drivers, including workload allocation, workspace design, and evaluation metrics. Technological practices address the configuration of the digital environment itself, distinguishing task-aligned from task-irrelevant tools. Assessment practices address how cognitive output is measured and credited. Table 1 summarises representative interventions at each level together with the evidence supporting them. Each of the strategies presented in Table 1 is supported by empirical research, with implementation approaches derived from randomized controlled trials, longitudinal studies, and validated workplace interventions as detailed in the supporting evidence column. The convergence of evidence across neuroscience, psychology, and organizational behaviour provides a robust foundation for these recommendations, elevating them beyond mere suggestions to evidence-based practices with measurable outcomes. The plasticity paradox identified in Section 3 is directly relevant here: the same neuroplastic adaptability that permits expert cognition to be acquired also consolidates the fragmented attentional profile elicited by habitual digital interruption. The multi-level strategies in Table 1 are therefore best understood as interventions that reverse maladaptive plasticity, not simply as productivity tools.

While our discussion has examined scientific research broadly, these principles apply across all disciplines, including medicine, social sciences, computer science, and engineering. The universal neurobiological mechanisms underlying deep thinking and the ubiquitous nature of digital distractions create common challenges that transcend disciplinary boundaries. Institutions could establish these strategies to cultivate conditions that facilitate researchers in sustaining concentration, minimizing interruptions, and nurturing profound, contemplative thinking.

### 7.2. Quantifying and Valuing Thinking Time in Scientific Research

Conventional performance measures frequently fail to recognize the significance of contemplation and generating ideas, resulting in an underestimation of the time spent on thinking in scientific research [80]. Contemporary employment procedures seldom measure the time spent on thinking, making it challenging to acknowledge and safeguard this crucial element of scientific research [81]. To address this issue, it is necessary to develop novel metrics and evaluation systems that quantify and value the cognitive processes that inspire scientific discovery, moving beyond publication counts to more nuanced measures of scientific impact and innovation [16]. As an illustrative starting point, protected thinking time could be operationalised as the cumulative daily duration of uninterrupted, single-task cognitive engagement. This could be logged via validated experience-sampling methods or passive sensing of device interaction patterns, combined with periodic self-report of task depth and output quality. Composite indicators derived from such data, capturing both the quantity and subjective quality of interruption-free intervals, would permit empirical testing of the hypothesised relationship between protected thinking time and research outcomes [16,17]. These methods should acknowledge that significant advancements arise frequently from deep thought and reflection rather than consistently measured outcomes [82]. Institutions and funding bodies, though often challenged by the intangible nature of thinking time, should consider re-evaluating their models to recognize and value the essential role of profound, contemplative work [83]. While quantifying reflective time presents methodological challenges, developing robust metrics that capture its contribution to research quality and innovation—such as those proposed in the Science of Science framework by Fortunato et al. [12]—could fundamentally transform how academic institutions and sporting organisations value and support impactful scientific work [83].

It is essential to conduct interdisciplinary research to examine the effects of time lost due to lack of concentration on the quality and substance of scientific work [16,17]. Cross-sectional and longitudinal studies should offer valuable insights into the associations between allocated periods of uninterrupted thinking and the quality of research, for the benefits of individuals, programs, and organisations. Indeed, researchers need to assess if these tools truly increase available cognitive capacity or rather lead to the opposite outcomes [84].

### 7.3. The Enduring Advantages of Giving Priority to Dedicated Periods of Thinking

The long-term institutional case for protecting thinking time rests on evidence that reflection supports creativity, transdisciplinary integration, and critical analysis prior to publication [51,85,86]. Institutions that embed this value structurally, rather than relying on individual discretion, will potentially be positioned to produce more impactful research.

### 7.4. Expertise as a Moderator

The relationship between protected thinking time and cognitive output is unlikely to be uniform across levels of domain expertise. Expert cognition is characterised by chunked schemata and rapid pattern recognition, which may reduce the wall-clock time required for certain integrative judgments while still requiring uninterrupted cognitive engagement [87]. Novices, by contrast, may need longer protected intervals to construct and consolidate the schemata that experts already possess; at the same time, novices may exhibit greater openness to divergent associations and suffer less from rigid mental sets. The optimal duration and structure of protected thinking time may therefore differ between expert and novice researchers, and time–quality relationships should be interpreted with this moderation in view. Empirical work specifying the expertise–time interaction is a priority for future research.

## 8. Conclusions

Our review addressed three questions posed in the Introduction section. First, regarding the neurobiological mechanisms through which task-irrelevant digital interruption impairs deep thinking, converging evidence indicates that repeated engagement with fragmented digital stimuli strengthens cortico-striatal circuits tuned to rapid switching while reducing consolidation of the prefrontal-parietal networks that support integrative cognition [6,26]. This plasticity paradox, whereby the same adaptive property that enables expertise also entrenches the attentional profile produced by the dominant digital environment, underlies the cognitive costs documented throughout our review. Second, regarding the conditions that constitute protected thinking time, the present synthesis identifies coordinated engagement of the executive control network and the default mode network as the neurobiological signature of deep thinking, and uninterrupted, interruption-free intervals as the temporal precondition for that engagement. These conditions are threatened not only by involuntary attentional capture from notifications but also by the voluntary offloading of substantive cognitive work to generative AI systems, a qualitatively distinct mechanism that warrants deliberate critical governance in nowadays research practice. Third, regarding evidence-based strategies, the multi-level framework summarised in Table 1 and Figure 1 spans individual, team, organisational, technological, and assessment dimensions, and its recommendations are empirically supported, though direct evidence linking protected thinking time to specific research-impact outcomes remains limited. Candidate institutional interventions that warrant controlled evaluation include designated deep-work periods, asynchronous communication norms, and evaluation metrics that weight conceptual innovation alongside publication volume. Expertise level is likely to moderate the protected-thinking-time–output relationship, and future empirical work should address this interaction. Research institutions that recognise protected thinking time as a neurobiological precondition rather than a discretionary workplace preference are most-probably better positioned to produce scientific work that is both rigorous and transformative.

## Figures and Tables

**Table 1:** Evidence-based strategies for promoting protected thinking time in research.

Strategy Level	Key Components	Implementation Approaches	Supporting Evidence
Individual time management	• Deep work periods • Cognitive optimization • Digital boundaries	• Schedule 90-min focused work blocks • Use Pomodoro Technique (25-min focus, 5-min break) • Implement digital detox periods • Practice daily mindfulness meditation	• Focused work blocks may improve cognitive performance on complex tasks (Newport [14]). • Time management techniques may enhance problem-solving in knowledge workers (Hanna [60]). • Digital boundaries are associated with reduced stress markers in longitudinal studies (Kawakami et al. [61]). • Daily meditation is linked to improved attentional control (Norris et al. [62]).
Physical and mental wellness	• Active rest periods • Sleep hygiene • Exercise integration	• Structured movement breaks • Maintain 7–9 h sleep schedule • Engage in regular physical activity per WHO recommendations • Practice mindfulness before creative tasks	• Walking breaks have been shown to improve creative thinking relative to seated conditions in controlled studies (Oppezzo & Schwartz [63]). • Optimal sleep duration and quality are associated with enhanced creative problem-solving and memory consolidation (Walker and Stickgold [54]). • Regular physical activity enhances neuroplasticity and is associated with increased BDNF levels (Mandolesi et al. [64]; Dinoff et al. [65]). • Pre-task mindfulness can improve divergent thinking (Wieth & Zacks [66]).
Team-based practices	• Communication protocols • Collaborative arrangements • Protected time blocks	• Implement asynchronous communication systems • Establish response-time expectations • Designate meeting-free days • Use shared “deep work” calendar blocking	• Asynchronous communication can reduce workday interruptions (Bernstein & Turban [67]). • Clear response-time agreements may decrease stress in research teams (Perlow et al. [68]). • Meeting-free days are associated with increased deep work output in knowledge workers (Burzynska and Stolarski [69]). • Calendar blocking can increase the completion of deep work tasks (DeFilippis et al. [70]).
Organizational framework	• Institutional policies • Environmental design • Support systems	• Implement transparent workload management • Create distraction-free zones and quiet rooms • Provide specialized professional development • Develop wellness support programs	• Transparent evaluation practices support research quality and institutional decision-making (Woolston [71]). • Distraction-free work environments have been associated with increased focus duration and reduced interruption frequency Ward et al. [72]. • Specialized cognitive training may improve research output quality (Shrout & Rodgers [73]). • Wellness programs show significant reductions in burnout symptoms among healthcare (West et al. [74]).
Technology integration	• Digital tools • AI optimization • Productivity systems	• Utilize focus-enhancement applications • Implement AI for routine task automation • Deploy screen time management tools • Monitor digital wellness metrics	• Notification-blocking interventions are associated with reduced attention fragmentation and improved task completion in workplace settings (Mark et al. [75]). • AI task automation may reduce cognitive load in research workflows (Tshitoyan et al. [76]). • Screen management tools are linked to reduced attention fragmentation (Ward et al. [72]). • Digital wellness monitoring can improve work–life boundaries (Roffarello & De Russis [77]).
Assessment and monitoring	• Performance metrics • Impact evaluation • Quality indicators	• Develop scientometric approaches beyond publication count • Implement qualitative output assessment • Track research translation and impact • Conduct regular cognitive wellness reviews	• Balanced metrics are associated with improved research innovation (Fortunato et al. [12]). • Qualitative assessment can increase research depth (Hicks et al. [78]). • Impact tracking is linked to improved research translation (Greenhalgh & Papoutsi [27]). • Regular cognitive wellness monitoring may reduce researcher burnout (Lovakov et al. [79]).

BDNF = Brain-Derived Neurotrophic Factor; RCTs = Randomized Controlled Trials; AI = Artificial Intelligence; WHO = World Health Organization.

## Data Availability

Not applicable.

## Abbreviations

ACT	Australian Capital Territory
AI	Artificial Intelligence
BDNF	Brain-Derived Neurotrophic Factor
DMN	Default Mode Network
EEG	Electroencephalography
fMRI	Functional magnetic resonance imaging
RCTs	Randomized Controlled Trials
REM	Rapid Eye Movement
WHO	World Health Organization
